# The use of natural infochemicals for sustainable and efficient harvesting of the microalgae *Scenedesmus* spp. for biotechnology: insights from a meta-analysis

**DOI:** 10.1007/s10529-016-2192-2

**Published:** 2016-08-26

**Authors:** Sebastiana Roccuzzo, Andrew P. Beckerman, Jagroop Pandhal

**Affiliations:** 1 Department of Chemical and Biological Engineering, University of Sheffield, Mappin Street, Sheffield, S1 3JD UK; 2 Department of Animal and Plant Sciences, University of Sheffield, Alfred Denny Building, Western Bank, Sheffield, S10 2TN UK

**Keywords:** Colony formation, *Daphnia* infochemicals, Harvesting biomass, Microalgae, Open raceway ponds, *Scenedesmus* spp

## Abstract

Open raceway ponds are regarded as the most economically viable option for large-scale cultivation of microalgae for low to mid-value bio-products, such as biodiesel. However, improvements are required including reducing the costs associated with harvesting biomass. There is now a growing interest in exploiting natural ecological processes within biotechnology. We review how chemical cues produced by algal grazers induce colony formation in algal cells, which subsequently leads to their sedimentation. A statistical meta-analysis of more than 80 studies reveals that *Daphnia* grazers can induce high levels of colony formation and sedimentation in *Scenedesmus obliquus* and that these natural, infochemical induced sedimentation rates are comparable to using commercial chemical equivalents. These data suggest that natural ecological interactions can be co-opted in biotechnology as part of a promising, low energy and clean harvesting method for use in large raceway systems.

## Introduction

Microalgal biotechnology has great promise arising from the diverse range of products that can be obtained from algal biomass, including fuels, animal feed, cosmetics, chemicals, nutraceuticals and pharmaceuticals. However, for low-to-medium value products (e.g. fuels, animal feed), large scale cultivation costs can make the process economically prohibitive (Gong and Jiang 2011). Currently, the most economic and practical cultivation method for large scale production of algal biomass for low-to-mid value products, is the raceway pond system, which has relatively low capital cost and can be relatively easy to operate and manage. However, the concentration of biomass within raceway ponds is usually low (0.5–1 kg m^−3^; Borowitz 2005) and therefore harvesting requires a large volume of water to be removed. Harvesting in this system can account for up to 30 % of the total production costs (Mata et al. 2010). Low energy, cost saving and easy-to-scale harvesting methods such as sedimentation and flotation are considered the most appropriate for these production systems. Both currently rely on pre-treatment stages to overcome the negative cell surface charge of algal cells referred to as coagulation and flocculation, essentially the induced clumping of algae.

### Challenges

Several surfactants or inorganic flocculants, such as FeCl_3_ or Al_2_(SO_4_)_3_, are used effectively to form large flocs, which rapidly settle to the reactor bottom, thus facilitating efficient harvesting. However, high dosages are usually required (80–250 mg l^−1^) (Shelef et al. 1984) and these can contaminate the biomass with metal hydroxide compounds. This can interfere with the final product application (i.e. human food additives or animals feed), downstream processing of the biomass, such as lipid extraction, or final product quality, including the oxidative stability of fuels and overall engine performance (Vandamme et al. 2013; Farooq et al. 2015). Furthermore, they can actually increase metal concentrations to toxic levels within the culture medium, a serious problem if water medium recycling is desirable for the process (Uduman et al. 2010). So while there are commercial chemical flocculants available, sustainable alternatives that do not impact on downstream processing or biomass use are needed and efforts have focussed on inducing autoflocculation of algal cells (Shen et al. 2015) as well as exploiting microbially produced bioflocculants (Oh et al. 2001).

Another challenge for open raceway pond algal cultivation is invasion from the surrounding environment by undesirable organisms. These include competing algal species, protozoa, viruses, bacteria, fungi and algae predators, or grazers, like the zooplankton rotifers, ciliates, copepods and cladocerans. These grazers are considered the highest risks (Bacellar and Vermelho 2013) and, in particular, cladocerans are considered to be the most problematic, as their large body size coupled with a high population growth rate can result in a rapid depletion of algal biomass. Zooplankton grazing of microalgae is a major issue for open raceway pond production of algal biomass and products (Pandhal and Noirel 2014). Yet, zooplankton grazers/algae ecology offers a way to view them as beneficial and links directly to the search for more natural flocculants (Lampert et al. 1994; Montemazzani et al. 2015).

### Potential applications of natural infochemicals

Infochemicals are chemical cues excreted by organisms that may change the behaviour, physiology and structure of individuals from another species. Many algal species respond to the presence of zooplankton grazers with inducible defences, a classic example of phenotypic plasticity (Hessen and van Donk 1993; Lürling 2003a). Chemical cues from the zooplankton can trigger changes in growth and development in algae leading to the formation of spikes and spines or changes in cell wall structure that lead to reduced consumption or palatability. These infochemicals could be exploited in large-scale biotechnology applications to reduce grazer activity and protect the biomass stock. Some research suggests that the infochemicals may be aliphatic sulfates and sulfamates (Yasumoto et al. 2008).

Many algal species also form aggregates or colonies in response to these chemical cues. Such colonies are too big for grazers to consume and induce algae sinking so that the algae are not accessible to the grazers (Hessen and van Donk 1993; Lürling, and van Donk 1996; Tollrian and Harvell 1999; Lürling 2003b). Although the metabolic and energetic costs associated with colony formation remain unclear, there is no doubt that they affect algal growth rates (Lürling and van Donk 1997; Zhu et al. 2015a). This colony formation defence has similarities with the aggregation response due to cell surface charge suppression with chemical flocculants.

### Scenedesmus spp. *responses*

A well-studied system of colony formation response to infochemicals includes the green algae within the genus *Scenedesmus*. *Scenedesmus* spp. are among the most commonly cultivated microalgae within open raceway ponds, possessing a tolerance to a wide range of environmental conditions. A single *Scenedesmus* genotype has the ability to produce one or more alternative morphologies in response to environmental conditions, including zooplankton grazing. The majority of studies investigating this phenomenon have focused on induced colony formation by chemical cues produced by the cladoceran zooplankton, *Daphnia* (Hessen and van Donk 1993; Lampert et al. 1994; Lürling 1999a, b, 2003a; van Holthoon et al. 2003; Mayeli et al. 2004; Pohnert et al. 2007; O’Donnell et al. 2013; Wu et al. 2013; Zhu et al. 2015a). Importantly, these responses can be generated solely via culture media that previously had *Daphnia* feeding on the algae (Lürling and van Donk 1997). This culture media can rapidly cause a shift of *Scenedesmus* population from predominant unicells to 2- 4- and 8- celled colonies (Hessen and van Donk 1993; Lampert et al. 1994; Lürling 1999a, b). Several features of these *Secendesmus* colonies are relevant to flocculation. First, colonies have higher sinking rates than unicells and, generally, sinking velocities increase with an increased colony size (Zhu et al. 2015b). Second, the magnitude of inducible colony formation can vary with duration of exposure to grazer cues and on grazer density. A number of studies have shown a positive correlation between *Daphnia* density and the extent of induced colony formation in *S. obliquus* (Lampert et al. 1994; Lurling and van Donk 1997; Lürling 1998; Wu et al. 2013).

### Biotechnology solutions

In a large-scale open cultivation system, infochemicals could be potentially used to induce colony formation and therefore bioflocculation (Montemazzani et al. 2015). These chemical cues are also expected to be abundant in zooplankton-rich ponds and therefore the outflow of water from raceways contaminated with grazers could be filtered to remove zooplankton grazers and algae, and then re-circulated into the pond to boost colony formation, prior to subsequent harvesting (Montemazzani et al. 2015). However, most work on predator induced algal colony formation has been undertaken from an ecology or evolutionary biology perspective. We change that by exploring, via a quantitative review, a wide range of data on algae response to cladoceran grazers, specifically evaluating these responses in the context of biotechnology applications.

### Use of infochemicals in industrial biomanufacturing

We address several specific issues related to the industrial potential of *Daphnia* spp. infochemicals. First, natural cues may be highly species-specific and even strain/genotype specific. It is important to uncover any specificity as this could impact on strain selection for industrial biomanufacturing. Second, the effect of size of grazer cues has never been estimated which would then allow standardised comparison among various grazers and, importantly, with the effects of chemical flocculants. Finally, the underlying mechanism of colony formation is still poorly understood; a systematic review facilitates insight into these mechanisms by synthesizing several metrics of colony size, including cell number and overall floc size. A more comprehensive understanding of the mechanisms involved would lead to improved process control during algal cultivation.

Our review cuts across several disciplines: data-reporting methods, experimental conditions and importantly, the strain/genotype/species identity of grazer and algae. Our quantitative synthesis provides insight into the intra- and inter-specificity of algae (*S. obliquus*)/grazer (*Daphnia* spp.) interactions associated with the production of colonies and a comparison between the effect size of biological cues and the effect size of commercially available chemicals.

## Methods

We searched Web of Science, StarPlus, Google Scholar, JStor and Mendeley databases with no constraint on publication year, using the following search term combinations: algae OR microalgae OR *Scenedemus* spp. OR *S. obliquus* OR *Chlorophyceae* OR *Scenedesmaceae* AND induced defences AND colony OR colony formation OR *coenobia* formation OR flocculation OR flocs OR aggregates OR morphology OR phenotypic plasticity OR mean particle volume AND grazers OR *Daphnia* OR *Daphninids* OR *Daphnia magna* OR *Cladocerans* OR chemical cues OR chemical signals OR infochemicals OR kairomones. This resulted in an initial set of ~70 papers which were further screened, so that studies focusing on the impact of algae properties on grazers or those without replicates were excluded. When not readily available or clearly reported, data were extracted from graphs by use of *WebPlotDigitizer*, a web based tool to obtain quantitative data from plots, images and maps. When necessary, authors were asked to provide either raw data or relevant information (e.g. mean, standard deviation, sample size) when data could not be directly extracted from papers. Studies could not be included if estimates of variation and sample size were unavailable.

### Effect size estimation

We estimated effect sizes in the form of standardized mean difference, SMD, using the Cohen’s d index. This is defined as “the unbiased standardized mean difference between an experimental group and its control” (Scheiner and Gurewitch 2001) and it is calculated as the difference between the experimental and control mean-s divided by the pooled standard deviation, corrected if necessary by a factor accounting for small sample size. We conducted a random-effects meta-analysis using R (R Core Team 2015) and the package *Metafor* (Viechtbauer^2010^). In every study, SMD was calculated from the difference between a treatment with infoche-micals or flocculant and a control, represented by algae only.

### Hypotheses

We first tested the Grazer Specificity hypothesis that species identity of cladoceran grazers will induce differential responses in the same algae species/strain. We then tested the Algae Specificity hypothesis, where for a single species of grazer (*D. magna*), we asked whether different strains of the same algae species respond differently to the same grazer infochemical. We also evaluated the hypotheses that (a) grazer feeding duration; (b) incubation time of grazer and algae together and (c) the grazer density used to produce the infochemicals, affected algae colony formation. Finally, in order to explore the potentialities of grazers’ cues in algal biotechnology, we examined whether *Daphnia* infochemicals can induce comparable responses in *Scenedesmus* to two chemical surfactants: FFD-6 (a surfactant solution made of 55 % water and 45 % mono- and didodecyl disulphanated diphenyloxide, sodium salt) and sodium dodecylsulfate (SDS) (Lürling and Beekman 2002; Lürling 2006).

## Results

After screening for standard meta-analytic criteria (sample size, mean and standard deviations reported), our data set comprised nine studies and 85 trials. Studies document effects of sevaral cladoceran grazers: *Daphnia pulicaria, D. pulex, D. magna, D. cucullata, D. galeata, D. galeata x hyalina and Ceriodaphnia reticulata.* The *S. obliquus* strains represented were UTEX 78, UTEX 1450, UTEX 2630, SAG 276/3A, SAG 276/1 and NIVA CHL6.

### Grazer specificity

Five studies provided 46 trials to compare the response of the mean particle volume (MPV) of *S. obliquus*, strain SAG 276/3A to infochemicals produced by seven grazer species. MPV was measured using a coulter counter and uses electrical impedance to measure the volume of particles as they pass individually through an aperture of defined size. In all studies, data were obtained by using filtered (0.1−0.2 μm) water sourced from tanks where individuals were allowed to graze on algae for 24 h **(**Table 1). Filtrate water was added in alls studies at concentrations between 4 and 10 % v/v.

**Table 1:** Range of grazer densities under study

Grazer	Individuals l^−1^
*D. pulicaria*	2, 5, 10, 20
*D. pulex*	50, 100, 200
*D. magna*	5, 10, 20, 50, 100, 200
*D. galeata x hyalina*	2, 5, 10, 20, 40
*D. galeata*	50, 100, 200
*D. cucullata*	50, 100, 200
*C. reticulata*	4, 10, 20, 40, 80, 160

**Fig. 1:**
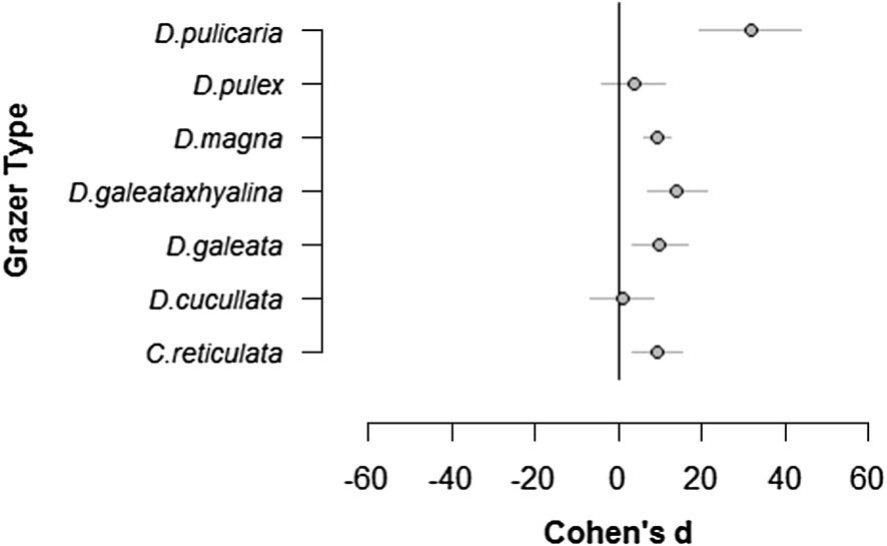
The effect of grazer (*Daphnia* spp.) identity on mean particle volume (MPV) of *S. obliquus,* strain SAG276/3A. Data are mean ± 95 CI of Cohen’s d, estimated from a random effects meta-analytic model of the effect of grazing after 2 days of exposure

We found that chemical cues in water from grazing *Daphnia* spp increased the MPV of *S. obliquus*, strain SAG 276/3A (Q(df) = 45, p < 0.001). We also detected grazer specificity (Fig. 1; Table 2); specifically, *D. pulicaria* produced the strongest effect, one that was double the average effect size of all other grazers. *D. magna, D. galeata, D. galeata x hyalina* and *C. reticulata* all induced colony formation at a moderate effect size. The effects of *D. pulex and D. cucullata* could not be distinguished from the control.

**Table 2:** Results of a random effects meta-analytic model of the effect of grazing

Type of grazer	Cohen’s d*	Lower 95 % CI	Upper 95 % CI
*D. pulicaria*	31.75	19.52	43.99
*D. pulex*	3.58	−4.01	11.18
*D. magna*	9.32	6.04	12.60
*D. galeata x hyalina*	14.08	6.92	21.23
*D. galeata*	9.92	3.26	16.57
*D. cucullata*	0.88	6.60	8.37
*C. reticulata*	9.31	3.42	15.21

* Size estimation was by using Cohen’s d index (see Methods)

### Algae strain specificity

**Fig. 2:**
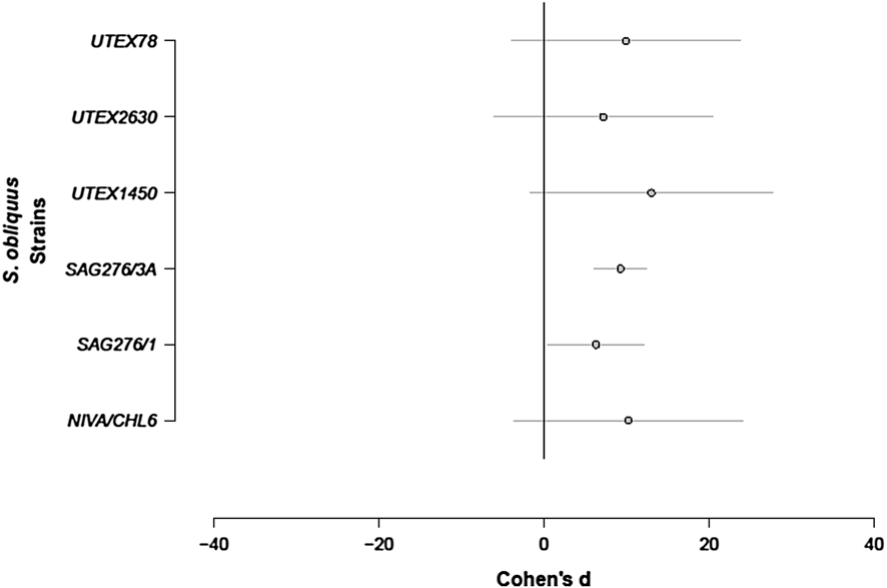
The change in mean particle volume (MPV) of six strains of *S. obliquus* exposed to filtered water from *D. magna* cultures. Data are mean ± 95 CI of Cohen’s d, estimated from a random effects meta-analytic model of the effect of grazing

Five studies providing 29 trials allowed us to compare the response of various strains of *S. obliquus* to infochemicals produced by *Daphnia magna*. We found that filtered *D. magna* water induced larger MPV overall (Fig. 2; Q (df = 28) = 189.9879, p < .0001). There were no significant differences among the strains (Fig. 2; omnibus p = 0.9424).

### Starvation, duration of incubation and density of grazers

**Fig. 3:**
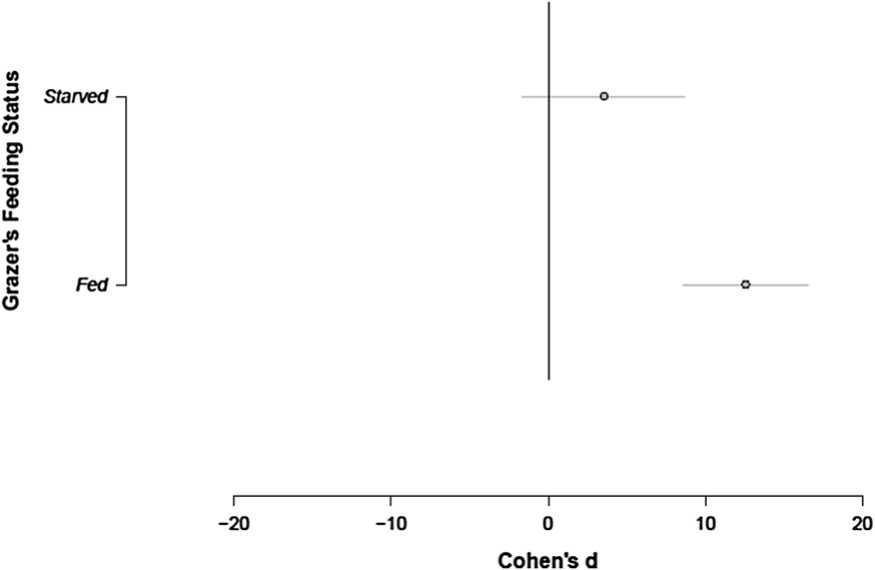
The effect of *Daphnia magna* food intake or starvation on mean particle volume of *S. obliquus*, strain SAG 276/3A. Data are mean ± 95 CI of Cohen’s d, estimated from a random effects meta-analytic model of the effect of grazing feeding status

Data for comparing the effects on algal MPV where *Daphnia magna* grazers were fed or starved were sourced from two studies with six trials with *infochemicals* from starved animals and seven studies with 50 trials for fed individuals. Time of exposure and grazers’ density effects were evaluated with data from seven studies and 56 trials. We found that water filtered from fed animals increased MPV (d = 12.5655, CI (8.5666;16.5645), but the effect of starved animals was highly variable (n = 6 trials) and could not be distinguished from zero *(*Fig. 3, d = 3.5318, CI (−1.6293; 8.6929).

We found that for *D. magna* there were no differences associated with 1,2 or 3 days of exposure to infochemicals (p-val = 0.8646) as well as no differences due to culture densities used to produce the infochemicals (p-val = 0.7374).

### Effect size comparison

**Fig. 4:**
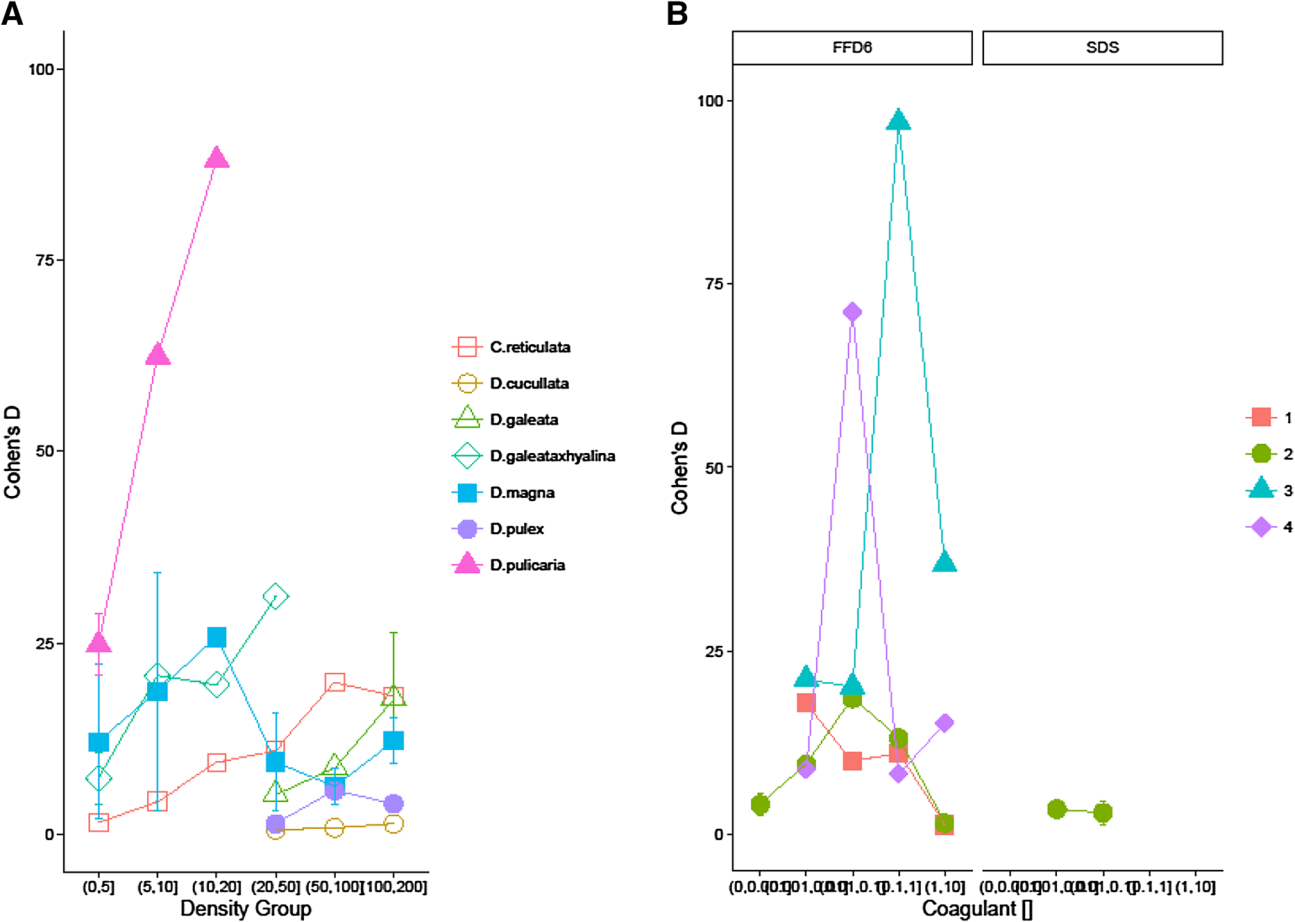
Comparison and contrast of mean effect sizes of *S. obliquus* mean particle volume induced variation, as affected by grazers culture density (**a**) and surfactants concentration levels (**b**)

We found a strong concentration dependent effect of both grazer *(*Fig. 4a) and surfactants (Fig. 4b). *D. pulicaria* produces double the effect size of the other grazers, and does so at dramatically lower densities (5–20 animals per litre). Furthermore, comparing the effect sizes of *D. pulicaria* with surfactants FFD-6 and SDS (Fig. 4b) shows that grazer infochemicals can rival or even outperform induced changes in MPV caused by the commercially available surfactants. We emphasize that the grazer data is for 2-day trials thus several grazer species produce effect sizes of similar or much greater magnitude (e.g. *D pulicaria*) in half the incubation time of FFD-6.

## Discussion

Our objective was to quantitatively evaluate the potential for cladoceran grazer infochemicals to induce colony formation, a phenomenon which might be exploited for microalgae flocculation and hence, biomass harvesting for biotechnology. We specifically addressed whether grazer species identity altered colony formation (grazer specificity) and whether different *S. obliquus* strains responded differentially to a common grazer (algae specificity). It was important to understand the specificity of colony formation as it entails an additional trait for selecting algal strains for large scale cultivation in biomanufacturing (addition to productivity, growth rates, resistance to diseases etc.), ultimately impacting on downstream processing. We also evaluated, via standardised effect sizes, whether grazer infochemicals generated effects at all similar to commercially available chemical surfactants, FFD-6 and SDS. Our findings suggest that cladoceran infochemicals show substantial promise: we found a significant effect of grazer identity, an effect size similar, or even higher under certain conditions, than commercial surfactants and no differences related to algae strains differentiation. However, data available were surprisingly constrained. Out of >70 possible papers, only nine studies with 85 trials offered data in a format to be included in the meta-analysis. Such low reporting rates of variation (e.g. standard deviation) and of sample sizes clearly hinders our ability to identify what appears to be a potentially positive use of infochemicals in industry.

### *Specificity and* Dephinia pulicaria

One of the most surprising outcomes associated with our assessment of grazer specificity was that the most commonly used species here, *D. magna*, reported in more than 50 % of the published papers, is relatively poor at inducing cell volume change. Instead, the relatively little studied *D. pulicaria,* appears able to produce *infochemicals* with the largest effect size, doubling the average of all the other grazers under study during the same incubation time (48 h; Fig. 4a). We also emphasize the capacity of *D pulicaria* to induce changes in particle volume which was not only higher than all other grazers, but generated these responses at very low culture densities, suggesting high promise. We must however note the small amount of data, requiring much more research. In addition to the standout effects of *D. pulicaria*, several other species “outperformed” the commonly cultured *D. magna*, *D. galeata x hyalina* also shows promise with a steadily rising effect on MPV that may continue to escalate at higher culture density (Fig. 4a).

### Infochemicals as novel algal flocculants

The advantage of using natural infochemicals over traditional coagulants, which neutralise the electrical charge of algal cells in water causing them to clump, include potentially lower costs, a more sustainable and environmentally friendly production process and reduced contamination of the growth media and feedstock. Although a comparison to traditional coagulants was not a motive in this meta-analysis, it was possible to calculate the standardisation of effect size and assess whether natural infochemicals can induce changes similar to that of commercially available surfactants. Figure 4a, B strongly suggest that infochemicals from more than one species have the potential to generate effects on the same scale as FFD-6 and well beyond SDS.

## Conclusions

This meta-analysis suggests the next steps from both an engineering and biotechnology perspective: designing methods to provide infochemical rich water for harvesting algal biomass that may be centred on recirculation of *Daphnia* cues medium. A potential biochemical agenda of identifying the chemical composition and species specificity of the infochemicals and ultimately their capacity for synthesis within an integrated system is highlighted. This is the first quantitative assessment of the importance of microalgae-grazers species-specific interactions and findings disclose the potential for developing an integrated bio-flocculation system based on natural infochemicals in open raceway ponds.
